# Comparative long-term outcomes of pembrolizumab plus chemotherapy versus pembrolizumab monotherapy as first-line therapy for metastatic non-small-cell lung cancer: a systematic review and network meta-analysis

**DOI:** 10.3389/fimmu.2024.1375136

**Published:** 2024-07-11

**Authors:** Shibo Huang, Zhilong Huang, Xiaolong Huang, Raoshan Luo, Weiming Liang, Tian Qin

**Affiliations:** The First Affiliated Hospital of Guangxi University of Science and Technology, Guangxi University of Science and Technology, Liuzhou, Guangxi, China

**Keywords:** pembrolizumab, chemotherapy, non-small-cell lung cancer, metastatic, meta-analysis

## Abstract

**Introduction:**

This systematic review and network meta-analysis(NMA) was designed to compare the long-term outcomes of pembrolizumab monotherapy and pembrolizumab plus chemotherapy as first-line therapy for metastatic non-small-cell lung cancer(NSCLC).

**Materials and Methods:**

Four databases(Medline, Embase, Web of Science and CENTRAL were searched published from establishment of database to August 17, 2023, for articles studying pembrolizumab monotherapy or pembrolizumab plus chemotherapy for non-small cell lung cancer (NSCLC). Network meta-analyses of progression-free survival(PFS), overall survival(OS), objective response rate(ORR), treatment-related adverse events(trAEs) and immune-related adverse events(irAEs) were performed.

**Results:**

A total of five studies were considered for NMA. This NMA includes a cohort of 2878 patients diagnosed with advanced NSCLC. Among them, 791 patients received pembrolizumab monotherapy, 1337 patients received chemotherapy, and 748 patients received pembrolizumab plus chemotherapy. The IPDformKM software was utilized to reconstruct Kaplan-Meier curves for OS and PFS, offering a lucid and intuitive depiction of oncological outcomes. For patients who have high levels of programmed death-ligand 1(PD-L1) expression (≥50%), pembrolizumab plus chemotherapy was more effective than using pembrolizumab alone as first-line therapy in terms of PFS (median survival time: 10.41 months versus 7.41 months, HR: 0.81, 95%CI 0.67 to 0.97, P=0.02) and ORR (RR:1.74, 95% CI: 1.25-2.43). Nevertheless, there was no statistically significant difference observed between the two groups in terms of OS (median survival time: 22.54 months versus 22.62 months, HR: 0.89, 95%CI 0.73 to 1.08, P=0.24). Furthermore, pembrolizumab plus chemotherapy provided a more advantageous long-term survival advantage in terms of OS (median survival time: 20.88 months versus 13.60 months, HR: 0.77, 95%CI: 0.62 to 0.95, P=0.015) compared to pembrolizumab monotherapy in patients with low PD-L1 expression levels (1% to 49%). With regards to safety, there was no statistically significant disparity between the two groups in relation to any irAEs (RD=0.02, 95% CI: -0.12 to 0.16) or Grade≥ 3 irAEs (RD=0.01, 95% CI: -0.10 to 0.12). Nevertheless, pembrolizumab plus chemotherapy exhibited a greater likelihood of encountering any trAEs (RD=0.23, 95% CI: 0.17 to 0.30) and Grade≥ 3 trAEs (RD=0.28, 95% CI: 0.21 to 0.35) in comparison to pembrolizumab monotherapy.

**Conclusions:**

The present network meta-analysis reported comparative long-term outcomes of pembrolizumab plus chemotherapy versus pembrolizumab monotherapy as first-line therapy for metastatic non-small-cell lung cancer. Pembrolizumab plus chemotherapy led to improved PFS and ORR in patients with advanced NSCLC who had a PD-L1 expression level of 50% or above. However, there was no noticeable benefit in terms of OS when pembrolizumab was paired with chemotherapy compared to utilizing pembrolizumab alone. In addition, pembrolizumab plus chemotherapy offered a greater long-term survival benefit in terms of OS when compared to utilizing pembrolizumab alone in patients with PD-L1 expression levels ranging from 1% to 49%. Furthermore, the increased effectiveness of pembrolizumab plus chemotherapy was accompanied by an increase in adverse side effects.

**Systematic review registration:**

https://www.crd.york.ac.uk/prospero/, identifier CRD42024501740.

## 1 Introduction

Lung cancer is the predominant form of cancer globally and the primary cause of mortality connected to cancer, resulting in about 1.7 million fatalities annually (1). About 80%-85% of lung cancers are pathologically classified as non-small cell lung cancer (2). Currently, surgery is the main approach used to treat early-stage non-small cell lung cancer. Nevertheless, a significant proportion of individuals experience the formation of either local or distant metastases (3). The therapeutic advancement of immune checkpoint blockade has significantly transformed the approach to treating and predicting outcomes for individuals diagnosed with NSCLC (4). Tumor cells possess many methods to resist immune system attacks, including the expression of immunosuppressive molecules on their cell surface, secretion of immunosuppressive substances, and recruitment of other immune cell populations with suppressive properties (5). Specific inhibitors against checkpoint receptors can block this immunosuppression, thereby increasing the specific immune response of T lymphocytes and eliciting an antitumor response (6, 7). Pembrolizumab is a humanized monoclonal antibody targeting programmed death 1 (PD-1), which has been shown to have antitumor activity in advanced non-small cell lung cancer (NSCLC). Single-agent pembrolizumab as first-line therapy is approved for tumors with high expression of PD-L1 (≥50%) while immunotherapy and chemotherapy are approved for any PD-L1 (8).

Pembrolizumab has demonstrated encouraging outcomes in recent clinical studies, particularly in cases where PDL1 staining is equal to or greater than 50% of tumor cells (9). Clinical trials and meta-analyses have shown that this treatment regimen can, in some cases, significantly improve patients’ overall survival and progression-free survival, while having a low toxicity profile (10). Additionally, the utilization of pembrolizumab in conjunction with chemotherapy has garnered considerable interest. The benefit of this combo treatment is its ability to achieve a wider range of effectiveness in patients with limited PD-L1 expression. Combining multiple therapies may offer longer-lasting disease management and improved survival advantages compared to using a single medication. Nevertheless, it is crucial to consider the potential toxicities and medication resistance associated with it (11). The KEYNOTE-042 study was an open label phase II-III randomized trial comparing pembrolizumab monotherapy with chemotherapy in the treatment of advanced NSCLC. The findings indicated that pembrolizumab outperformed chemotherapy in terms of overall survival and progression-free survival in the overall population. Moreover, there were more substantial enhancements in OS and PFS specifically for the subset of tumors with PDL1≥50% (12). The KEYNOTE-189 study was a randomized phase III trial that evaluated the efficacy of pembrolizumab in combination with chemotherapy compared to chemotherapy alone for the treatment of advanced NSCLC. The findings demonstrated that the combination of pembrolizumab and chemotherapy outperformed chemotherapy alone in terms of OS and PFS in both the overall population and the subgroup of tumors with a PDL1≥50% expression (13).

However, there is currently a lack of clinical trials of chemotherapy combined with pembrolizumab versus pembrolizumab monotherapy to determine whether chemotherapy combined with pembrolizumab has a higher benefit than pembrolizumab monotherapy in metastatic non-small cell carcinoma. Network meta-analysis enables the comparison of treatment arms in randomized controlled trials (RCTs) by utilizing relative and absolute measures of treatment efficacy and common treatment arms (14–16). Prior network meta-analyses (17–19) had shown the effectiveness and safety of combining pembrolizumab with chemotherapy compared to using pembrolizumab alone. However, since the original articles only presented short-term outcomes, they failed to provide information on the long-term outcomes of RCTs. Over the course of the last three years, multiple randomized controlled trials (RCTs) have updated their long-term outcomes (13, 20–23). Hence, it is both possible and essential to perform a meta-analysis that compares the long-term outcomes of pembrolizumab plus chemotherapy versus pembrolizumab monotherapy as the initial treatment for metastatic non-small-cell lung cancer.

The aim of our study was to indirectly compare the long-term outcomes of pembrolizumab in combination with chemotherapy versus pembrolizumab monotherapy as first-line therapy. We additionally assessed the disparities in survival rates among patients with tumors exhibiting PD-L1 expression ranging from 1% to 49%.

## 2 Material and methods

### 2.1 Search strategy

The present meta-analysis was performed in accordance with the 2020 standards of the Preferred Reporting Project for Systematic Review and Meta-Analysis (PRISMA).This study has been registered at PROSPERO with a registration number of CRD42024501740. Four databases including of PubMed, Embase, Web of science, and the Cochrane Library were systematically searched for literatures published up to August 17, 2023, and a combination of MeSH and free-text words were searched according to the PICOS principle, using the following searching strategy: (“pembrolizumab” AND “Chemotherapy” AND “Non Small Cell Lung” AND “randomized controlled trial”). **Supplementary Material 1** presented the searching record in detail.

### 2.2 Inclusion and exclusion criteria

Inclusion criteria were as follows (1): Comparing pembrolizumab versus chemotherapy, or pembrolizumab plus chemotherapy versus chemotherapy, or pembrolizumab versus pembrolizumab plus chemotherapy; (2) Untreated metastatic non-small-cell lung cancer; (3) Median follow-up time was at least 48 months, and at least one of the following outcomes were reported: PFS, OS, Grade≥ 3 irAEs rate, Grade≥ 3 TRAEs rate; (4) Randomized controlled trials.

Exclusion criteria: (1) Other types of articles, such as case reports, letters, reviews, meta-analyses, editorials, animal studies and protocols; (2) Not RCTs; (3) Unable to extract data; (4) Reduplicate cohort of patients.

### 2.3 Selection of studies

The selection of research, including duplicate removal, was managed using EndNote (Version 20; Clarivate Analytics). Two reviewers independently conducted the initial search, eliminated duplicate records, evaluated the titles and abstracts for relevance, and categorized each study as either included or omitted. We reached a resolution by achieving consensus. In the absence of a consensus, a third review author assumed the role of an arbitrator.

### 2.4 Data extraction

Two reviewers independently extracted the data. Data retrieved included patient groups and numbers, age, sex, smoking status, Eastern Tumor Cooperative Group(ECOG), brain metastases, histological type, PD-L1 TPS, the name of the study, first author, year of publication, ORR, OS, PFS, trAEs, Grade≥ 3 trAEs, irAEs, Grade≥ 3 irAEs, Kaplan-Meier curves for OS, Kaplan-Meier curves for PFS. Discrepancy was resolved by consulting with a third investigator.

### 2.5 Risk of bias assessment

**Figure 1:**
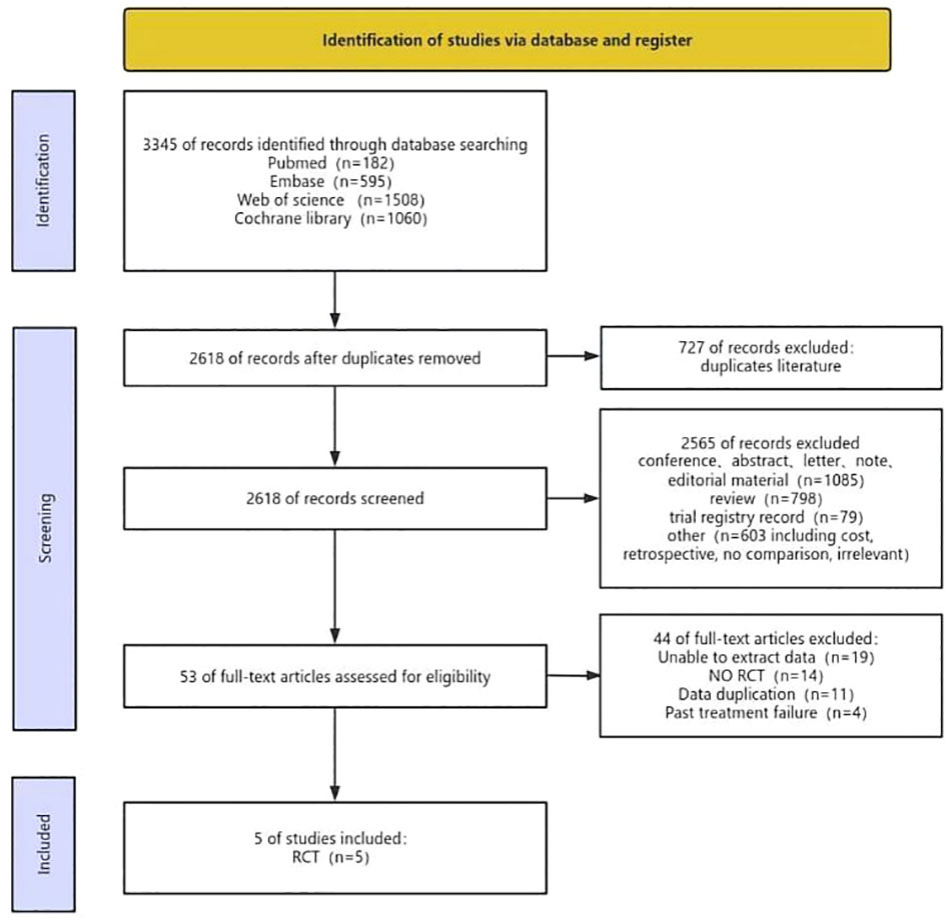
Flow chart of literature search strategies.

The risk of bias in the trials included was assessed by two independent reviewers using the Cochrane Risk of Bias tool, according to the following domains: random sequence generation, allocation concealment, blinding of participants and personnel, blinding of outcome assessment, incomplete outcome data, selective reporting and others bias. If there were discrepancies, the controversial results were resolved by group discussion. The quality evaluation of the literature is shown in **Figure 1**.

### 2.6 Statistical analysis

The selection duplicate removal of studies included was conducted using EndNote (Version 20; Clarivate Analytics). Review manager 5.3(Cochrane Collaboration, Oxford, UK), Stata 12.0, Statistical software R (version 4.3.1, https://www.r-project.org/), the R package “netmeta” and “IPDformKM” package were used for data analysis (24). We quantified Kaplan-Meier curves for RFS and OS using GetData Graph Digitizer software and reconstructed individual data through the IPDformKM package. Individual patient-level data were reconstructed using the method established by Guyot et al. (25). Upon reconstructing the individual patient data, the patients were categorized into groups. Patients who received chemotherapy were assigned to the chemotherapy cohort, while patients who received pembrolizumab monotherapy were assigned to the pembrolizumab cohort. Another cohort was formed consisting of patients who had pembrolizumab in combination with chemotherapy. Subsequently, we recreated the survival curves for the three groups in order to gain insight into long-term survival following treatment with three distinct interventions. All the results were analyzed by random effects model. P value < 0.05 was considered statistically significant.

## 3 Results

### 3.1 Search results

**Figure 2:**
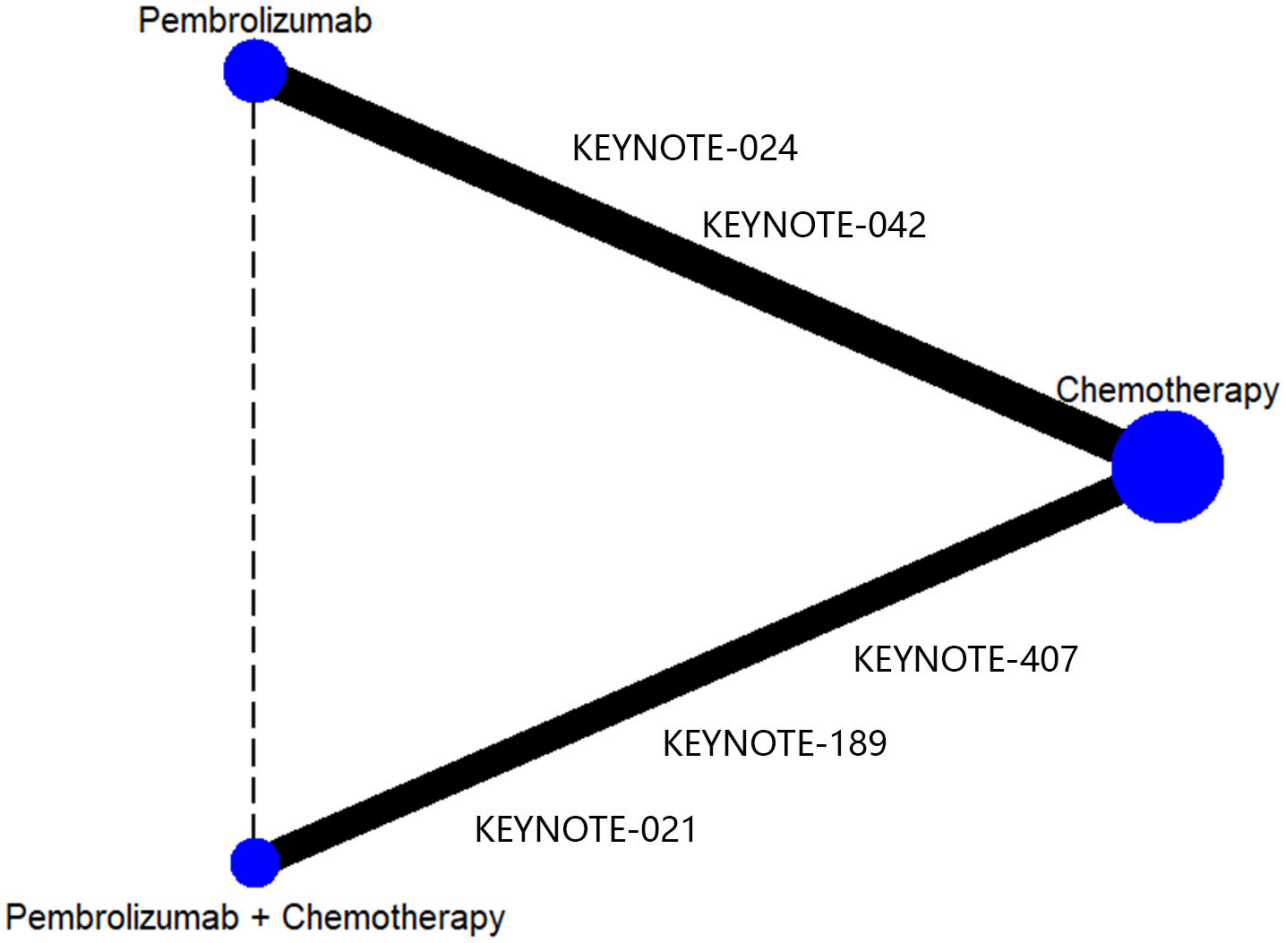
Network diagram of indirect comparison.

After doing the initial search, a total of 3345 publications were identified. However, after removing duplicate research, only 2618 cases remained. Out of these papers, a total of 2565 were eliminated from consideration after evaluating the titles and abstracts. Ultimately, a total of 53 articles were accessible for a comprehensive examination of their complete content. Following the application of the inclusion criteria, 5 trials were chosen for inclusion. Two of these evaluated pembrolizumab monotherapy versus platinum chemotherapy (21, 22) and three evaluated pembrolizumab combined with platinum chemotherapy versus platinum chemotherapy (13, 20, 23). The detail process of inclusion and exclusion of literature is shown in **Figure 1**. Data from the included RCT trials were used to construct a network of RCTs that indirectly compared pembrolizumab plus chemotherapy versus pembrolizumab alone, with chemotherapy as the common control group (**Figure 2**).

### 3.2 Patient characteristics

This study includes a sample of 2878 patients diagnosed with metastatic NSCLC. Among them, 791 patients were randomly assigned to receive pembrolizumab monotherapy, 1337 patients were assigned to receive chemotherapy alone, and 748 patients were assigned to receive combination therapy consisting of pembrolizumab and chemotherapy. Though the specific chemotherapy regimens were different among five RCTs, all chemotherapy cohorts were administered platinum-based combination treatment (**Table 1**). The baseline features of the patients, such as age, ECOG performance status, smoking status, masculinity, brain metastases status, and previous treatment, were comparable. All chemotherapy cohorts were administered platinum-based combination treatment. **Table 1** displays the characteristics of the studies that were included.

**Table 1:** Characteristics of included studies and patients.

Study ID	KEYNOTE-024	KEYNOTE-189	KEYNOTE-407	KEYNOTE-042	KEYNOTE-021
	C (n = 151) P (n = 154)	C (n = 206) P + C (n = 410)	C (n = 281) P + C (n = 278)	C (n = 637) P (n = 637)	C (n = 63) P + C (n = 60)
Author/Year	Reck 2021	Garassino 2023	Novello 2023	Castro 2022	Awad 2020
Median follow-up time (months)	59.5	64.4	56.9	61.1	49.4
Comparator chemotherapy regimen	Carboplatin + pemetrexed or	Cisplatin + pemetrexed or	Carboplatin + paclitaxel or	Carboplatin + paclitaxel or	Carboplatin + pemetrexed
	Cisplatin + pemetrexed or	Carboplatin + pemetrexed	Carboplatin + nab-paclitaxel	Carboplatin + pemetrexed	
	Carboplatin + gemcitabine or				
	Cisplatin + gemcitabine or				
	Carboplatin + paclitaxel				
PD-L1 expression					
*>* 50% (%)	151 (100) 154 (100)	70 (34) 132 (32)	73 (26) 73 (26)	300 (47) 299 (47)	17(27) 20(33)
1–49% (%)	NA	58 (28) 128 (31)	104 (37) 103 (37)	337 (53) 338 (53)	23 (37) 19 (32)
*<* 1% (%)	NA	78 (38) 150 (37)	104 (37) 102 (37)	– –	23 (37) 21 (35)
Median Age (range)	66 (38–85) 65 (33–90)	64 (34–84) 65 (34–84)	65 (29–87) 65 (36–88)	63 (56–68) 64 (57–69)	63.2(58–70) 62·5 (54–70)
Male Sex (%)	95 (63) 92 (60)	109 (53) 254 (62)	220 (79) 235 (84)	204 (69) 210 (70)	26 (41) 22 (37)
ECOG PS (%)					
0	53 (35) 54 (35)	80 (39) 186 (45)	73 (26) 90 (32)	96 (32) 91 (30)	29 (46) 24 (40)
1	98 (65) 99 (65)	125 (61) 221 (55)	205 (74) 191 (68)	203 (68) 209 (70)	34 (54) 35 (58)
2	NA	0 1 (*<*1)	NA	NA	
Smoking Status (%)					
Current/Former	132 (87) 149 (97)	181 (88) 362 (88)	256 (92) 262 (93)	235 (79) 233 (78)	54 (86) 45 (75)
Never	19 (13) 5 (3)	25 (12) 48 (12)	22 (8) 19 (7)	64 (21) 67 (22)	9 (14) 15 (25)
Histology					
Squamous	27 (18) 29 (19)	NA	274 (98) 271 (98)	107 (36) 114 (38)	NA
Non-squamous	124 (82) 125 (81)	NA	NA	192 (64) 186 (62)	NA
Adenosquamous	NA	NA	7 (3) 6 (2)	NA	NA
Adenocarcinoma	NA	198 (96) 394 (96)	NA	NA	55 (87) 58 (97)
NSCLC NOS	NA	4 (2) 10 (2)	NA	NA	7 (11) 2 (3)
Other	NA	4(2) 6 (2)	6 (2) 19 (7)	NA	1 (2) 0(0)
Brain Metastases (%)	10 (7) 18 (12)	35 (17) 73 (18)	20 (7) 24 (9)	NA	7 (11) 12 (20)
Previous therapy (%)					
Neoadjuvant therapy	3 (2) 1 (1)	6 (3) 5 (1)	NA	1 (*<*1) 5 (2)	NA
Adjuvant therapy	6 (4) 3 (2)	14 (7) 25 (6)	5 (2) 8 (3)	8 (3) 4 (1)	5 (8) 4 (7)

C, chemotherapy; CI, confidence interval; ECOG PS, Eastern Cooperative Oncology Group performance status; HR, hazard ratio; NSCLC NOS, non-small cell lung cancer; p, pembrolizumab; PD-L1, programmed death ligand 1; PFS, progression-free survival; p + c, chemotherapy combined with pembrolizumab; OS, overall survival.

### 3.3 Risk of bias

**Figure 3:**
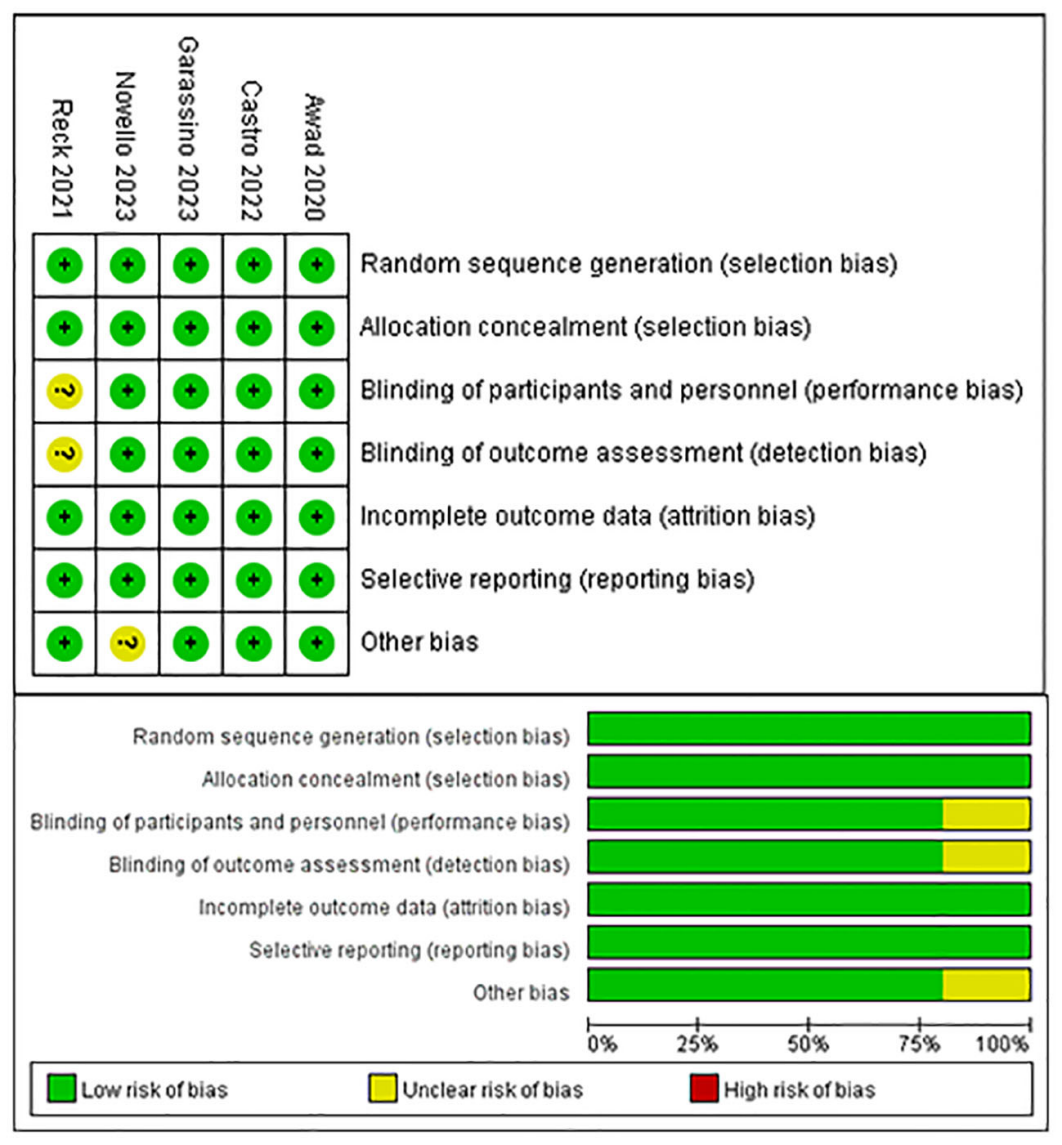
Risk of bias assessment diagram.

**Figure 3** provides a summary of the risk of bias assessment results. Among the 5 studies, an adequate randomized sequence was generated in five studies, appropriate allocation concealment was reported in five studies, the blinding of participants was clear in four studies, the blinding of outcome assessors was reported in four studies, outcome data were complete in five studies, five studies had no selective reporting, and four studies had no other bias.

### 3.4 Progression-free survival - PD-L1 TPS ≥ 50%

**Figure 4:**
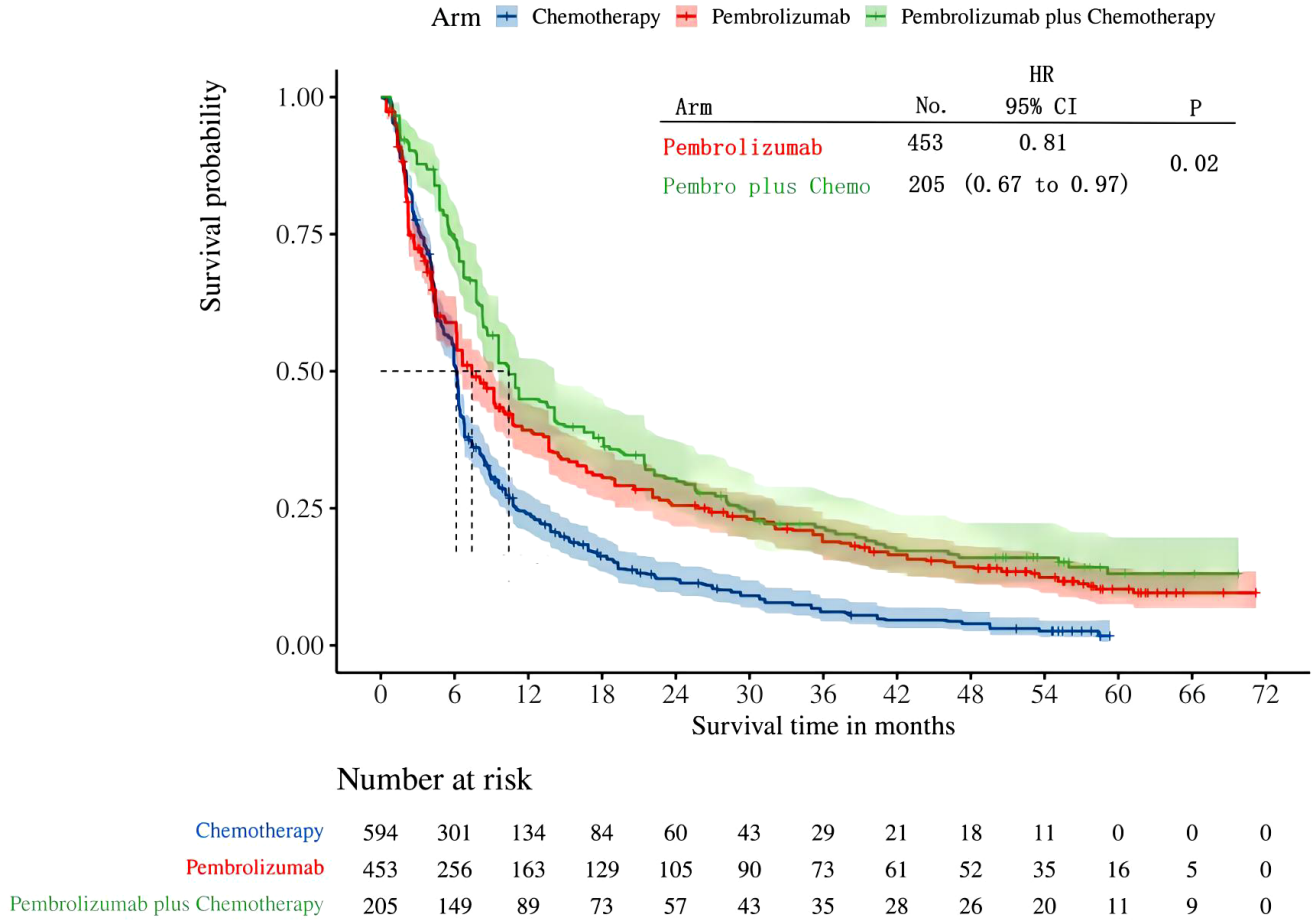
Kaplan-Meier curves for PFS in patients with PD-L1 TPS ≥ 50%.

Four studies provided data on PFS in patients with high levels of PD-L1 expression (≥50%). Among these studies, KEYNOTE-024 and KEYNOTE-042 reported PFS outcomes with pembrolizumab monotherapy, while KEYNOTE-407 and KEYNOTE-189 revealed PFS outcomes with pembrolizumab in conjunction with chemotherapy. Following the reconstruction of the cohort, we conducted a new analysis of PFS specifically in patients with a tumor PD-L1 expression level of 50% or higher. The Kaplan-Meier curve demonstrates that the combination of pembrolizumab with chemotherapy provides a superior long-term survival advantage compared to pembrolizumab alone in terms of PFS(HR: 0.81, 95%CI: 0.67 to 0.97, P=0.02) (**Figure 4**). The PFS median survival time for pembrolizumab combined with chemotherapy was 10.41 months, while it was 7.41 months for pembrolizumab monotherapy and 6.13 months for chemotherapy alone. We provided periodic updates on PFS of each group at 6-month intervals from 0 to 36 months, which are displayed in **Table 2**.

**Table 2:** Results of OS and RFS.

Outcomes	6 month	12 month	18 month	24 month	30 month	36 month	Median survival time
PD-L1 TPS≥50% PFS							
Pembro + chemo	73.82%	44.95%	37.87%	30.44%	23.21%	22.63%	10.41 month
pembrolizumab	59.11%	38.93%	30.97%	25.66%	22.87%	20.05%	7.41 month
chemotherapy	52.03%	23.71%	16.81%	12.21%	8.84%	6.85%	6.13 month
PD-L1 TPS≥50% OS							
Pembro + chemo	86.38%	70.18%	61.78%	48.74%	44.07%	41.62%	22.54 month
pembrolizumab	77.64%	66.46%	54.72%	46.88%	41.65%	35.11%	22.62 month
chemotherapy	74.30%	51.59%	40.41%	32.57%	26.97%	21.73%	12.93 month
PD-L1 TPS1-49% OS							
Pembro + chemo	85.84%	69.02%	55.22%	42.83%	35.39%	30.61%	20.88 month
pembrolizumab	71.32%	53.62%	45.13%	35.39%	23.36%	22.47%	13.60 month
chemotherapy	78.23%	51.32%	35.92%	27.43%	20.70%	16.46%	12.35 month

### 3.5 Overall survival - PD-L1 TPS ≥ 50%

**Figure 5:**
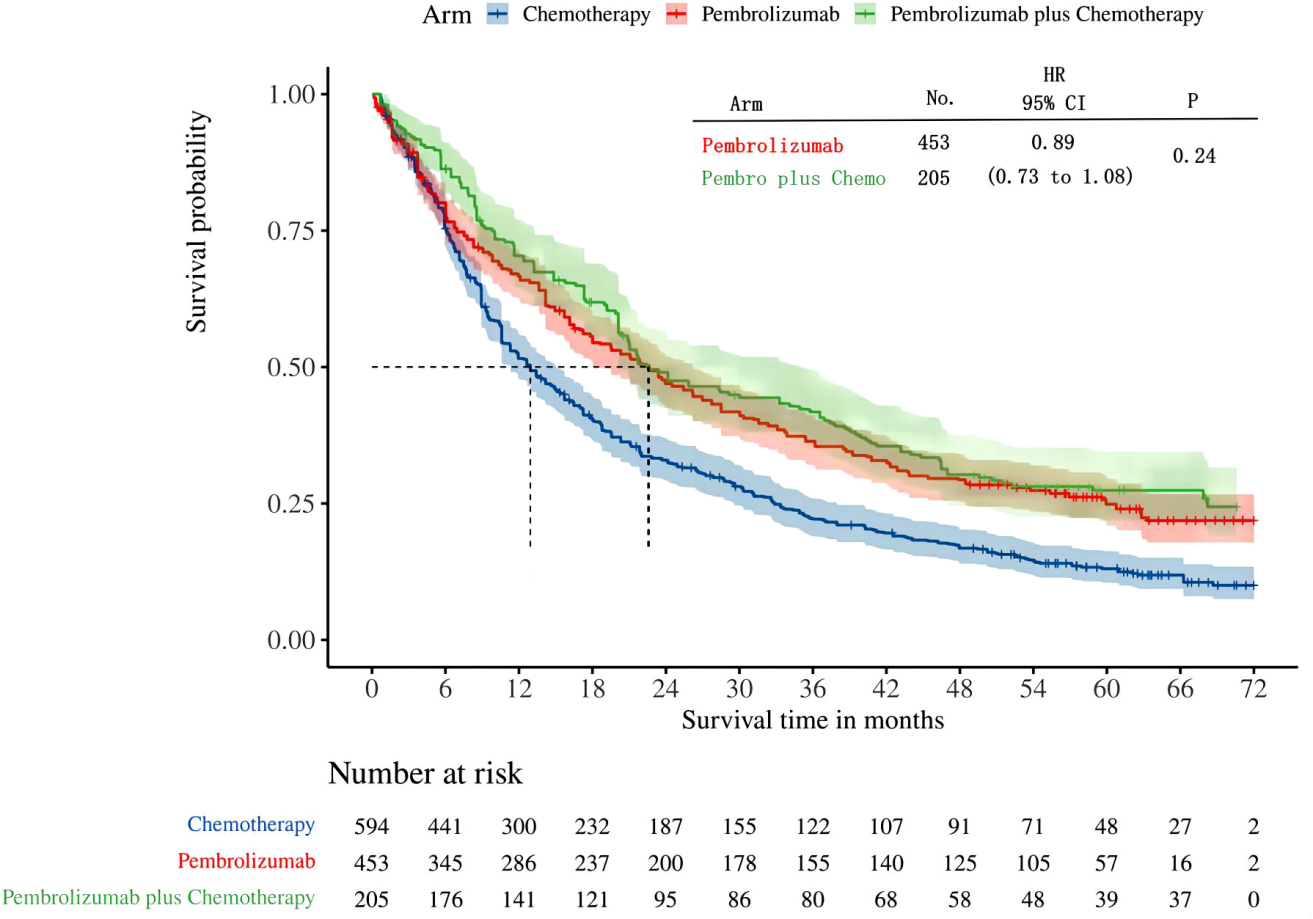
Kaplan-Meier curves for OS in patients with PD-L1 TPS ≥ 50%.

A total of four studies reported overall survival in patients with high levels of PD-L1 expression (≥50%). Among these studies, KEYNOTE-024 and KEYNOTE-042 reported OS with pembrolizumab monotherapy, and KEYNOTE-407 and KEYNOTE-189 reported OS with pembrolizumab in combination with chemotherapy. Following the reconstruction of the cohort, we conducted a new analysis of OS specifically in patients with a tumor PD-L1 expression level of 50% or higher. The Kaplan-Meier curve demonstrates that there was no statistical significance in terms of OS between two groups(HR: 0.89, 95%CI: 0.73 to 1.08, P=0.24) (**Figure 5**). The median survival time for OS was 22.54 months for the combination of pembrolizumab and chemotherapy, 22.62 months for pembrolizumab monotherapy, and 12.93 months for chemotherapy alone. We provided periodic updates on OS of each group at 6-month intervals from 0 to 36 months, which are displayed in **Table 2**.

### 3.6 Objective response rate–PD-L1 TPS > 50%

**Figure 6:**
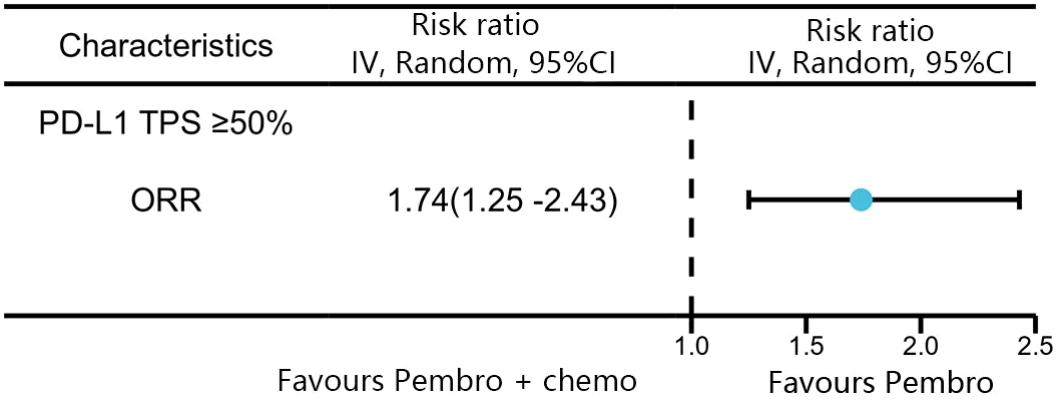
Network meta-analysis results for ORR in patients with PD-L1 TPS ≥ 50%.

A total of four studies reported ORR in patients with high levels of PD-L1 expression (≥50%). Among these studies, KEYNOTE-024 and KEYNOTE-042 reported ORR with pembrolizumab monotherapy, and KEYNOTE-407 and KEYNOTE-189 reported ORR with pembrolizumab in combination with chemotherapy. The network meta-analysis revealed that the combination of pembrolizumab with chemotherapy had a superior response rate compared to pembrolizumab alone in patients with high levels of PD-L1 expression (≥50%)(RR:1.74, 95% CI: 1.25-2.43) (**Figure 6**).

### 3.7 Overall survival - PD-L1 TPS 1%-49%

**Figure 7:**
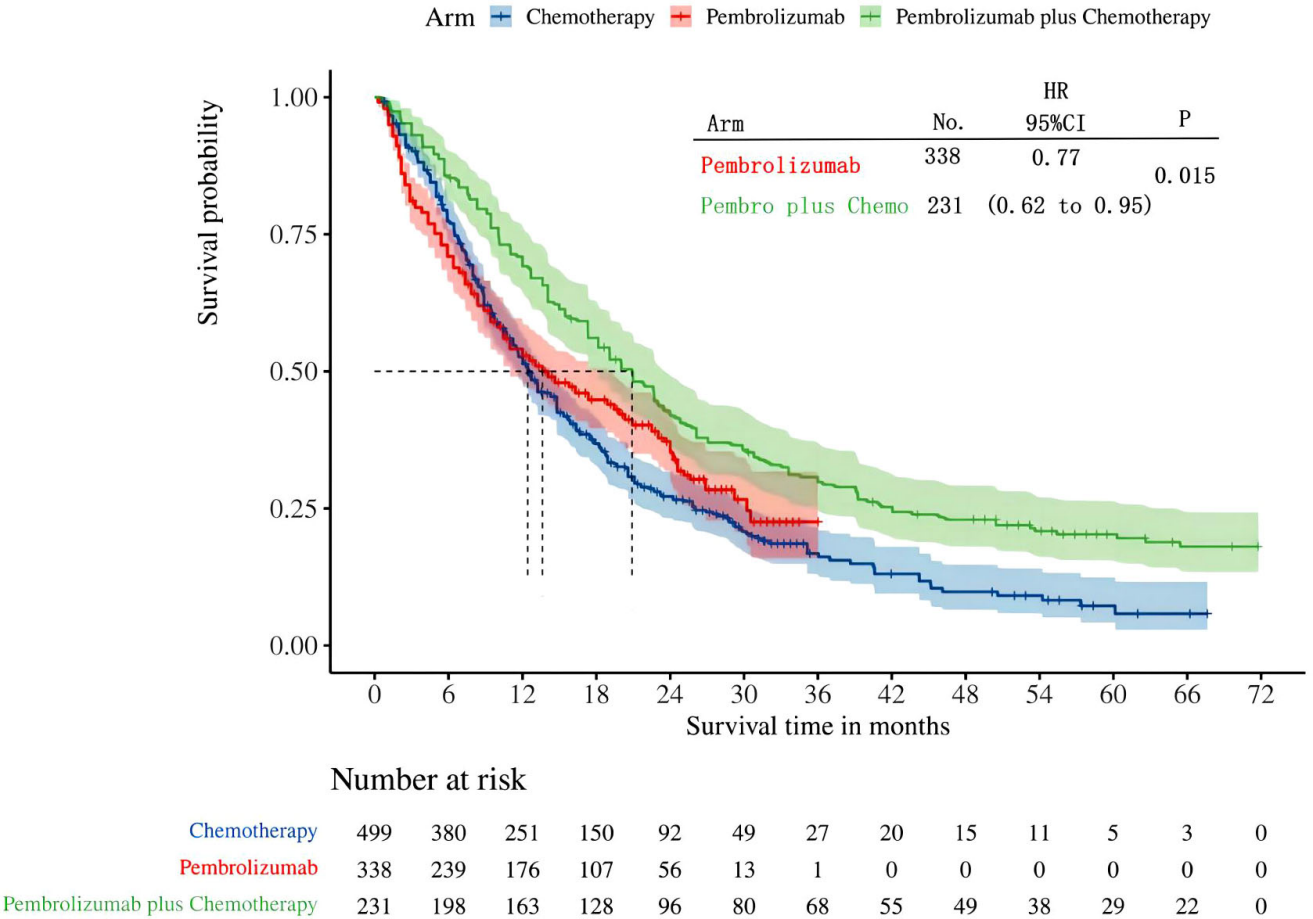
Kaplan-Meier curves for OS in patients with PD-L1 TPS 1-49%.

Three studies in all reported OS in individuals whose tumors expressed PD-L1 in the range of 1-49%. Among these studies, the KEYNOTE-042 trial presented OS data for pembrolizumab monotherapy, while the KEYNOTE-407 and KEYNOTE-189 trials presented OS for pembrolizumab plus chemotherapy. After reconstructing the cohort, we performed an updated evaluation of OS especially in patients with a tumor PD-L1 expression level of 1-49%. The Kaplan-Meier curves demonstrate that the combination of pembrolizumab with chemotherapy provides a superior long-term survival advantage compared to pembrolizumab alone in patients with tumor PD-L1 expression ranging from 1% to 49% (HR: 0.77, 95%CI: 0.62 to 0.95, P=0.015) (**Figure 7**). The median survival time for OS was 20.88 months for pembrolizumab plus chemotherapy, 13.60 months for pembrolizumab monotherapy, and 12.35 months for chemotherapy. We provided periodic updates on OS of each group at 6-month intervals from 0 to 36 months, which are displayed in **Table 2**. Insufficient relevant data prevented us from conducting a comparison of PFS between pembrolizumab and pembrolizumab plus chemotherapy.

### 3.8 Treatment-related adverse events

**Figure 8:**
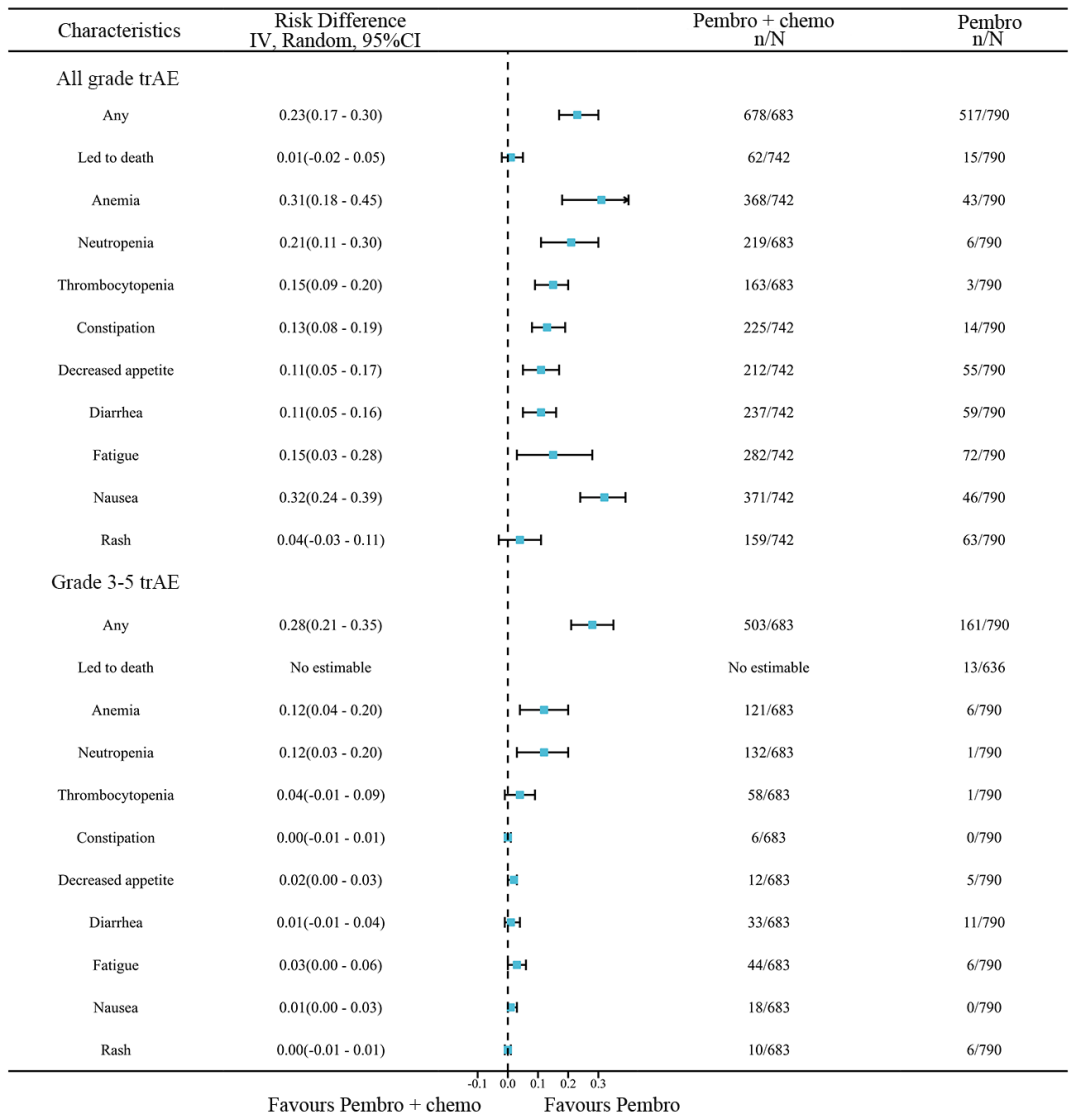
Network meta-analysis results for trAEs.

A network meta-analysis was conducted to compare any trAEs and Grade≥ 3 trAEs between pembrolizumab monotherapy and chemotherapy plus pembrolizumab (**Figure 8**). The meta-analysis results indicate that chemotherapy plus pembrolizumab was linked to a greater likelihood of any trAEs compared to pembrolizumab monotherapy(RD=0.23, 95% CI: 0.17 to 0.30). Chemotherapy combined with pembrolizumab had a greater occurrence of the following trAEs compared to pembrolizumab alone: mortality, anemia, neutropenia, thrombocytopenia, constipation, reduced appetite, diarrhea, fatigue, nausea, and rash. The meta-analysis findings suggest that the combination of chemotherapy and pembrolizumab is associated with a higher probability of Grade≥ 3 trAEs compared to pembrolizumab monotherapy(RD=0.28, 95% CI: 0.21 to 0.35). The combination of chemotherapy and pembrolizumab resulted in a higher incidence of the following Grade≥ 3 trAEs compared to pembrolizumab monotherapy: anemia and neutropenia.

### 3.9 Immune-related adverse events

**Figure 9:**
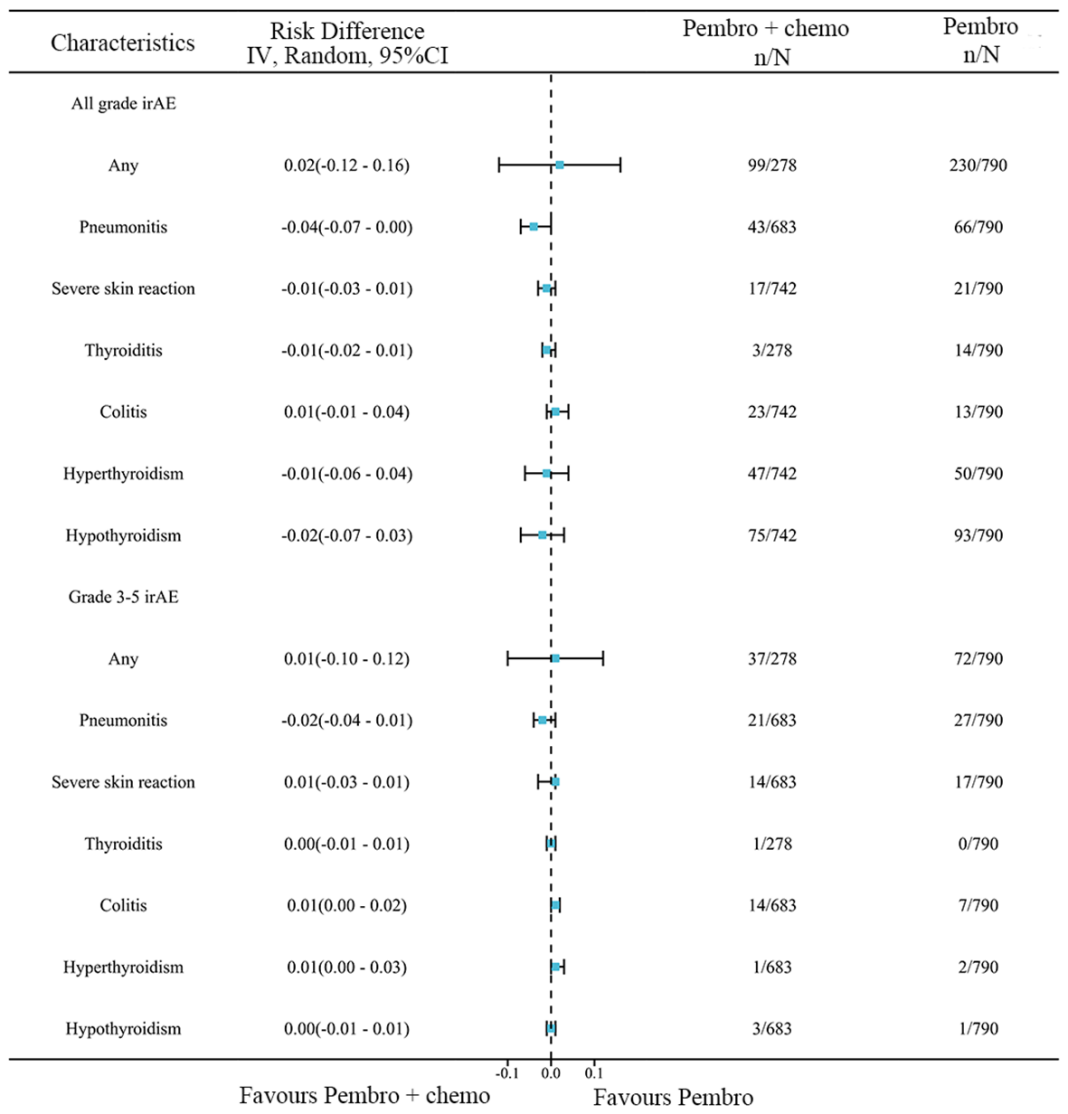
Network meta-analysis results for irAEs.

A network meta-analysis was performed to assess any irAEs and Grade≥ 3 irAEs between pembrolizumab monotherapy and chemotherapy plus pembrolizumab (**Figure 9**). The meta-analysis findings suggest that there was no statistically significant difference between the two groups in terms of any irAEs(RD=0.02, 95% CI: -0.12 to 0.16) or Grade≥ 3 irAEs(RD=0.01, 95% CI: -0.10 to 0.12).

## 4 Discussion

Activated T cells, B cells, natural killer cells, monocytes, and dendritic cells express programmed cell death receptor-1 (PD-1), a type I transmembrane glycoprotein belonging to the Ig superfamily (26). As an important immune checkpoint proteins, PD-1 interacts with two ligands, PD-L1 (B7-H1) and PD-L2(B7-H2), respectively. Immune and epithelial cells inductively express PD-L1, while antigen-presenting cells express PD-L2. In a physiological sense, PD-1 prevents immune system dysregulation by interacting with antigen-presenting cell surface PD-L1 and PD-L2. By overexpressing PD-L1, tumor cells encourage PD-1 binding to surface-expressed PD-L1 molecules, which in turn impairs immune surveillance of T cells, making it more difficult for tumor cells to be recognized and killed, and encouraging tumor immune escape (27). By disrupting interactions of PD-1/PD-L1, tumor immune tolerance can be broken, tumor specific T cells can regain their killing ability, and tumor clearance can be achieved by PD-1/PD-L1 monoclonal antibodies (28). Pembrolizumab, a PD-L1 inhibitor, has received approval for the treatment of NSCLC due to its notable clinical efficacy (29). The KEYNOTE-189 study was a randomized phase III trial that assessed the effectiveness of pembrolizumab in conjunction with chemotherapy in comparison to chemotherapy alone for treating advanced NSCLC. The results showed that the combination of pembrolizumab plus chemotherapy was more effective than chemotherapy alone in terms of OS and PFS in both the entire study population and the subset of tumors with a PDL1≥50% expression (13).

The results of our study offer robust evidence-based recommendations about the long-term prognosis for choosing between pembrolizumab monotherapy or pembrolizumab in combination with chemotherapy in clinical practice. Notably, the IPDformKM software was used to reconstruct Kaplan-Meier curves for OS and PFS, providing a clear and intuitive representation of oncological outcomes. Our results indicate that in patients with advanced NSCLC who have high levels of PD-L1 expression, the combination of pembrolizumab and chemotherapy is more efficacious than pembrolizumab alone as the first-line therapy in terms of PFS (median survival time: 10.41 months versus 7.41 months) and ORR (RR:1.74). However, there was no statistically significant distinction between the two groups in terms of OS. The notable enhancement in terms of ORR and PFS might be attributed to the stimulation of neoantigen release induced by chemotherapy, as well as the synergistic impact of immunotherapy and chemotherapy. However, the chemotherapy regimens are generally maintained for only three to four months(every 3 weeks, 4 to 6 cycles), while the pembrolizumab regimens are generally maintained for two years (every 3 weeks, 35 cycles). Over time, the residual effects of chemotherapy will progressively diminish, leaving only the lingering effects of pembrolizumab. In addition, pembrolizumab in combination with chemotherapy offers a more favorable long-term survival benefit in relation to OS (median survival time: 20.88 months versus 13.60 months) when compared to pembrolizumab monotherapy in patients with PD-L1 expression levels ranging from 1% to 49%. Since the data was not available, we could not provide the progression-free survival data among the PD-L1 TPS 1-49%. Regarding safety, there was no statistically significant difference between the two groups in terms of any irAEs or Grade≥ 3 irAEs. However, the combination of chemotherapy and pembrolizumab was associated with a higher probability of experiencing any trAEs and Grade≥ 3 trAEs compared to using pembrolizumab alone, suggesting that the enhanced effectiveness of pembrolizumab plus chemotherapy came with the drawback of increased adverse reactions.

Chemotherapeutics possess the capacity to enhance the immune system’s capability to identify and react to malignancies, or they can eliminate cells that inhibit the immune system. Furthermore, they have the potential to alter certain elements of the tumor microenvironment (30). It is crucial for us to distinguish these effects as we progress since chemotherapeutics have the ability to postpone the development of drug resistance, which could potentially change the chances of survival. Our meta-analysis consistently confirms that combining chemotherapy with first-line immune checkpoint medicines, such as pembrolizumab, enhances the efficacy of treatment for patients with advanced NSCLC. This phenomenon can be partially elucidated by the synergistic impact of immunotherapies and the induction of neoantigen release prompted by chemotherapy (31). There is variability among immune checkpoint inhibitors. Nivolumab and Pembrolizumab are immunosuppressive medications that inhibit the PD-1 protein, while Durvalumab and Atezolizumab were specifically engineered to target the PD-1, PD-L1 ligand (32). This approach has the potential to be used more extensively in order to reduce the impact on particular subgroups of NSCLC. However, further research is required. Genetic alterations of PD-L1 have been observed, which often result in the over-expression of PD-L1 (33). Xianhuo Wang et al. discovered that the genetic mutations of PD-L1 were specifically positioned in the exons of PD-L1 (34). These changes could impact the function of immunoglobulins and the transmembrane action of PD-L1, thus altering the immune response against tumors. Multiple studies have indicated that the expression of PD-L1, a protein associated with lung and other solid tumors, can be modified following treatment with platinum-based chemotherapy or concomitant chemoradiation (35). Fujimoto et al. conducted a study where they found that the expression of PD-L1 dropped dramatically after concurrent chemoradiation therapy in patients with locally advanced non-small lung cancer. This decrease in PD-L1 expression was linked to a positive prognosis (36). Toshiaki Takahashi et al. observed a considerable decrease in PD-L1 expression after treatment with pembrolizumab (37). This connection could potentially be one of the contributing factors to resistance against ICI and warrants additional exploration in extensive investigations.

An major strength of this study is that it is the first network meta-analysis to compare the long-term outcomes of pembrolizumab plus chemotherapy versus pembrolizumab monotherapy as first-line therapy for metastatic non-small-cell lung cancer. Previous network meta-analyses (17–19) exclusively reported outcomes that were limited to the short-term. Our findings support the existing scientific evidence about the long-term prognosis for pembrolizumab plus chemotherapy as first-line therapy for metastatic non-small-cell lung cancer. Besides, the Kaplan-Meier curves for OS and PFS were recreated to allow for a clear and comprehensible representation of the oncological outcomes.

This network meta-analysis possesses inherent limitations. The IPDformKM software was used to obtain reconstructed individual patient data from published KM curves of different quality. The quality of the analysis may be impacted. it is important to exercise caution when interpreting the results due to the potential for errors in reconstructing individual data. However, earlier research has demonstrated that HR obtained from rebuilt data has exhibited superior accuracy compared to published HR (38). Inconsistent baseline characteristics of patients in different clinical trials, such as doses and schedules of chemotherapeutic regimens, PD-L1 expression, gender, ECOG PS, smoking status, histology, metastases, and neoadjuvant therapy, may lead to heterogenicity in term of efficacy assessment and long-term survival assessment. Chemotherapy alone showed similar treatment effects in the five RCTs in terms of median PFS and OS (**Supplementary Material 2**), implying the that the types of chemotherapeutic agent would not lead to no obvious impact. By stratifying patients based on PD-L1 TPS, the heterogeneity caused by PD-L1 expression was minimized, leading to improved reliability in pooling the results. Unfortunately, this meta-analysis did not have access to data on individual patients, which means that it was not possible to conduct subgroup analysis based on other inconsistent baseline characteristics. To address these constraints, it is imperative to conduct controlled randomized trials to directly assess the effectiveness of pembrolizumab combination chemotherapy in comparison to pembrolizumab monotherapy.

In conclusion, the present network meta-analysis reported comparative long-term outcomes of pembrolizumab plus chemotherapy versus pembrolizumab monotherapy as first-line therapy for metastatic non-small-cell lung cancer. Pembrolizumab plus chemotherapy resulted in enhanced PFS and ORR among patients with advanced NSCLC who had a PD-L1 expression level of 50% or above. Nevertheless, there was no discernible advantage in terms of OS when pembrolizumab was combined with chemotherapy in comparison to using pembrolizumab alone. Furthermore, pembrolizumab plus chemotherapy provided a more advantageous long-term survival advantage in terms of OS compared to using pembrolizumab alone in patients with PD-L1 expression levels ranging from 1% to 49%. In addition, the heightened efficacy of pembrolizumab in combination with chemotherapy was accompanied by a rise in undesirable side effects.

## Data availability statement

The original contributions presented in the study are included in the article/**Supplementary Material**. Further inquiries can be directed to the corresponding authors.

## Author contributions

SH: Conceptualization, Data curation, Investigation, Software, Writing – original draft. ZH: Conceptualization, Data curation, Investigation, Software, Writing – original draft. XH: Data curation, Formal analysis, Writing – original draft. RL: Data curation, Formal analysis, Writing – original draft. WL: Funding acquisition, Methodology, Writing – review & editing. TQ: Funding acquisition, Methodology, Writing – review & editing.
