# Supplementary material for: Comparative long-term outcomes of pembrolizumab plus chemotherapy versus pembrolizumab monotherapy as first-line therapy for metastatic non-small-cell lung cancer: a systematic review and network meta-analysis
Source: Front Immunol. 2024 Jul 11;15:1375136. doi: 10.3389/fimmu.2024.1375136 (PMC11273333; doi:10.3389/fimmu.2024.1375136)
Supplement: Supplementary file 1 [file Table_1.docx]

| PubMed | | |
| --- | --- | --- |
| No. | Query | Results |
| 1 | ((((Pembrolizumab[Title/Abstract]) OR (SCH-900475[Title/Abstract])) OR (lambrolizumab[Title/Abstract])) OR (MK-3475[Title/Abstract])) OR (Keytruda[Title/Abstract]) | 8268 |
| 2 | ((((((((((("Carcinoma, Non-Small-Cell Lung"[Mesh]) OR (Carcinoma, Non Small Cell Lung[Title/Abstract])) OR (Carcinomas, Non-Small-Cell Lung[Title/Abstract])) OR (Lung Carcinoma, Non-Small-Cell[Title/Abstract])) OR (Lung Carcinomas, Non-Small-Cell[Title/Abstract])) OR (Non-Small-Cell Lung Carcinomas[Title/Abstract])) OR (Non-Small-Cell Lung Carcinoma[Title/Abstract])) OR (Non Small Cell Lung Carcinoma[Title/Abstract])) OR (Carcinoma, Non-Small Cell Lung[Title/Abstract])) OR (Non-Small Cell Lung Carcinoma[Title/Abstract])) OR (Non-Small Cell Lung Cancer[Title/Abstract])) OR (Nonsmall Cell Lung Cancer[Title/Abstract]) | 98466 |
| 3 | ((((((Chemotherapy[Title/Abstract]) OR (Therapy, Drug[Title/Abstract])) OR (Drug Therapies[Title/Abstract])) OR (Therapies, Drug[Title/Abstract])) OR (Chemotherapies[Title/Abstract])) OR (Pharmacotherapy[Title/Abstract])) OR (Pharmacotherapies[Title/Abstract]) | 497659 |
| 4 | (randomized controlled trial [pt] OR controlled clinical trial [pt] OR randomized [tiab] OR placebo [tiab] OR clinical trials as topic [mesh:noexp] OR randomly [tiab] OR trial [ti]) NOT (animals [mh] NOT humans [mh]) | 1431454 |
| 5 | 1 AND 2 AND 3 AND 4 | 182 |

| Web of science | | |
| --- | --- | --- |
| No. | Query | Results |
| 1 | TS=(Pembrolizumab OR SCH-900475 OR lambrolizumab OR MK-3475 OR Keytruda) | 19694 |
| 2 | TS=(Chemotherapy OR Therapy, Drug OR Drug Therapies OR Therapies, Drug OR Chemotherapy OR Chemotherapies OR Pharmacotherapy OR Pharmacotherapies) | 6134483 |
| 3 | TS=(Carcinoma, Non-Small-Cell Lung OR Carcinoma, Non Small Cell Lung OR Carcinomas, Non-Small-Cell Lung OR Lung Carcinoma, Non-Small-Cell OR Lung Carcinomas, Non-Small-Cell OR Non-Small-Cell Lung Carcinomas OR Non-Small-Cell Lung Carcinoma OR Non Small Cell Lung Carcinoma OR Carcinoma, Non-Small Cell Lung OR Non-Small Cell Lung Carcinoma OR Non-Small Cell Lung Cancer OR Nonsmall Cell Lung Cancer) | 148926 |
| 4 | TS=(randomized controlled trial OR controlled clinical trial OR Randomized OR placebo OR clinical trials as topic OR randomly OR Trial OR Prospective) | 3902636 |
| 5 | 1 AND 2 AND 3 AND 4 | 1508 |

| Embase | | |
| --- | --- | --- |
| No. | Query | Results |
| 1 | ‘pembrolizumab’:ti,ab,kw OR ‘SCH-900475’:ti,ab,kw OR ‘lambrolizumab’:ti,ab,kw OR ‘MK-3475’:ti,ab,kw OR ‘Keytruda’:ti,ab,kw | 18899 |
| 2 | 'carcinoma, non-small-cell lung'/exp OR 'carcinoma, non-small-cell lung' OR (('carcinoma,'/exp OR carcinoma,) AND 'non small cell' AND ('lung'/exp OR lung)) OR 'carcinoma, non small cell lung':ti,ab,kw OR 'carcinomas, non-small-cell lung':ti,ab,kw OR 'lung carcinoma, non-small-cell':ti,ab,kw OR 'lung carcinomas, non-small-cell':ti,ab,kw OR 'non-small-cell lung carcinomas':ti,ab,kw OR 'non-small-cell lung carcinoma':ti,ab,kw OR 'non small cell lung carcinoma':ti,ab,kw OR 'carcinoma, non-small cell lung':ti,ab,kw OR 'non-small cell lung carcinoma':ti,ab,kw OR 'nonsmall cell lung cancer':ti,ab,kw OR 'non-small cell lung cancer':ti,ab,kw | 225584 |
| 3 | 'chemotherapy'/exp OR chemotherapy OR 'therapy, drug':ti,ab,kw OR 'drug therapies':ti,ab,kw OR 'therapies, drug':ti,ab,kw OR chemotherapy:ti,ab,kw OR chemotherapies:ti,ab,kw OR pharmacotherapy:ti,ab,kw OR pharmacotherapies:ti,ab,kw | 1356336 |
| 4 | 'randomized controlled trial':ab,ti OR 'controlled clinical trial':ab,ti OR randomized:ab,ti OR placebo:ab,ti OR 'clinical trials as topic':ab,ti OR randomly:ab,ti OR trial:ab,ti OR prospective:ab,ti | 2976768 |
| 5 | 1 AND 2 AND 3 AND 4 | 1060 |

| cochrane library | | |
| --- | --- | --- |
| No. | Query | Results |
| 1 | (Pembrolizumab OR SCH-900475 OR lambrolizumab OR MK-3475 OR Keytruda):ab,ti,kw | 2853 |
| 2 | (Carcinoma, Non-Small-Cell Lung OR Carcinoma, Non Small Cell Lung OR Carcinomas, Non-Small-Cell Lung OR Lung Carcinoma, Non-Small-Cell OR Lung Carcinomas, Non-Small-Cell OR Non-Small-Cell Lung Carcinomas OR Non-Small-Cell Lung Carcinoma OR Non Small Cell Lung Carcinoma OR Carcinoma, Non-Small Cell Lung OR Non-Small Cell Lung Carcinoma OR Non-Small Cell Lung Cancer OR Nonsmall Cell Lung Cancer):ti,ab,kw | 15955 |
| 3 | (Chemotherapy OR Therapy, Drug OR Drug Therapies OR Therapies, Drug OR Chemotherapy OR Chemotherapies OR Pharmacotherapy OR Pharmacotherapies):ti,ab,kw | 561536 |
| 4 | (randomized controlled trial OR controlled clinical trial OR Randomized OR placebo OR clinical trials as topic OR randomly OR Trial OR Prospective):ti,ab,kw | 1469840 |
| 5 | 1 AND 2 AND 3 AND 4 | 595 |
