# Supplementary material for: Comparative long-term outcomes of pembrolizumab plus chemotherapy versus pembrolizumab monotherapy as first-line therapy for metastatic non-small-cell lung cancer: a systematic review and network meta-analysis
Source: Front Immunol. 2024 Jul 11;15:1375136. doi: 10.3389/fimmu.2024.1375136 (PMC11273333; doi:10.3389/fimmu.2024.1375136)
Supplement: Supplementary file 2 [file Table_2.docx]

| Trial | PFS, median(95%CI), months | OS, median(95%CI), months |
| --- | --- | --- |
| KEYNOTE-024 | 5.5 (4.2 – 6.2) | 13.4 (9.4 – 18.3) |
| KEYNOTE-042 | 6.5 (6.2 – 7.6) | 12.2 (10.4 – 14.6) |
| KEYNOTE-189 | 4.8 (3.1 – 6.2) | 10.1 (7.5 – 22.0) |
| KEYNOTE-407 | 4.2 (2.9 – 4.8) | 11.5 (7.5 – 17.1) |

Supplementary material 2. Median progression-free survival and overall survival of
chemotherapy arm in each study included in the meta-analysis.

Abbreviations: PFS, progression-free survival; OS, overall survival; CI, confidence interval.
